# *Blastomyces dermatitidis*: Antibody Detection in Sera from Dogs with Blastomycosis with Yeast Lysate Antigens Produced from Human and Dog Isolates

**DOI:** 10.1155/2014/376725

**Published:** 2014-02-27

**Authors:** Katie Mondada, Jessie Fullmer, Eric Hungerford, Katrina Novack, Kristen Vickers, Gene Scalarone

**Affiliations:** Department of Biological Sciences, Idaho State University, P.O. Box 8007, 921 S. 8th Avenue, Pocatello, ID 83209, USA

## Abstract

Dogs are common hosts to the fungal organism *Blastomyces dermatitidis*, which causes the systemic disease blastomycosis. The goal of our study was to compare the reactivity of two *B. dermatitidis* yeast lysate antigens prepared from dog isolates (ERC-2, Wisconsin; T-58, Tennessee) and two lysate antigens prepared from human isolates (B5931 and B5896, Minnesota) against 48 serum specimens from dogs with confirmed blastomycosis using the indirect enzyme-linked immunosorbent assay (ELISA). Secondarily, we used three different ELISA substrates (Ultra TMB: A, SureBlue: B, and SureBlue Reserve: C) to compare the effectiveness of each substrate. Mean absorbance values ranged from 0.446 (B) to 0.651 (C) for the B5931 antigen and from 0.393 (B) to 0.540 (C) for the ERC-2 antigen in Trial 1. In Trial 2, the absorbance values ranged from 0.628 (B) to 0.909 (A) for the B5896 antigen and from 0.828 (B) to 1.375 (C) for the T-58 antigen. In Trial 1, the lysate antigen prepared from the human isolate B5931 exhibited the highest absorbance value and in Trial 2 the lysate prepared from the dog isolate T-58 was the most reactive. The overall results thus indicated that the T-58 lysate was the optimal reagent when used to detect antibody with the Sure-Blue Reserve substrate. Our laboratory is continuing to study *B. dermatitidis* antigen and substrate combinations for the reliable immunodiagnosis of blastomycosis in humans and animals.

## 1. Introduction

Blastomycosis produced by *Blastomyces dermatitidis*, a systemic fungal infection of animals and humans, is a dimorphic organism that exists in nature as a filamentous (mycelial) form in soil and decomposing organic matter. Traditionally, the geographic distribution of blastomycosis has been associated with southeastern and south-central states that border the Ohio and Mississippi Rivers and upper Midwestern states including areas in Wisconsin and Minnesota, which are highly endemic for the disease. The fungus exists in environments with heavy incidences of humidity, fog, rain, and dew, which facilitate the spread of the mycelial spores [1, 2].

*B. dermatitidis* is present in the mycelial form in nature or in the laboratory at approximately 25°C and has the ability to convert to the yeast phase at 37°C in the lungs of the infected host. The disease may be self-resolving or it may exist as an acute or chronic state in the pulmonary tissue, where it may be misdiagnosed as tuberculosis or some other pulmonary disease. If the disease is not diagnosed or untreated while in the lungs, it may become invasive and disseminate to other organs including the skin, eyes (ocular involvement in dogs), bones, and, possibly, the central nervous system where fatal meningitis may develop [3–7].

The diagnosis of blastomycosis has presented clinicians with varying difficulties over the years. In some instances culturing or histopathological examination, which have been the gold standard for diagnosis, may be beneficial, but in some patients these methods may not yield the desired results due to the time required for culture growth or procedures involved in invasive testing. This has led to several investigators being involved in research designed to improve immunological assays, which tend to provide a more rapid diagnosis, but problems still exist with regard to the sensitivity and specificity of immunoassays [8–11]. A recent study [12] on the use of *B. dermatitidis *surface protein in an enzyme immunoassay for antibody detection in blastomycosis indicated a sensitivity value of 87.8% in patients with blastomycosis, specificity of 99.2% in patients with non-fungal infections and healthy subjects and 94.0% in patients with histoplasmosis.

Our laboratory has developed novel yeast phase lysate antigens and utilized these in various immunoassays for antibody detection in serum specimens from dogs and humans [13–18], but these studies have only opened up new avenues of approach with regard to how we might improve immunodiagnostic methods in the future. Therefore, this present study was designed to determine the reactivity of four *B. dermatitidis* lysate antigens when used in combination with three peroxidase substrates in the indirect ELISA to detect antibodies in serum specimens from dogs with diagnosed blastomycosis, as described below.

## 2. Materials and Methods

### 2.1. Antigens

*B. dermatitidis* yeast phase lysate reagents (B5931, human, Minnesota; ERC-2, dog, Wisconsin; B5896, human, Minnesota; and T-58, dog, Tennessee) were prepared following a protocol similar to the one used for the production of antigen from *Histoplasma capsulatum* [19–21] and modified in our laboratory for *B. dermatitidis* lysate antigen production [22]. The yeast phase cells were grown for 7 days at 37°C in a chemically defined medium (glucose, 10.0 g; potassium phosphate monobasic, 1.5 g; calcium chloride dehydrate, 0.15 g; magnesium sulfate, 0.5 g; ammonium sulfate, 2.0 g; L-asparagine, 2.0 g; L-cysteine, 0.2 g; and pH adjusted to 6.2 with 5 N sodium hydroxide) in an incubator shaker, harvested by centrifugation (700 ×g; 5 min) followed by washing with distilled water, resuspended in distilled water, and then allowed to lyse for 1 day at 37°C in water with shaking. The preparations were centrifuged, filter-sterilized, merthiolate-added (1 : 10,000), and stored at 4°C. Protein determinations were performed on the lysates using the BCA protein assay kit (Pierce Chemical Company, Rockford, IL, USA), and dilutions of the antigenic reagents used in the ELISA assays were based on protein concentration.

### 2.2. Serum Specimens

Forty-eight serum specimens from dogs with diagnosed blastomycosis and 10 serum specimens from normal, noninfected dogs were provided by Dr. A. M. Legendre (University of Tennessee College of Veterinary Medicine, Knoxville, TN).

### 2.3. ELISA

The ability of each *B. dermatitidis* yeast lysate reagent to detect antibodies in the above serum specimens was determined using the indirect enzyme-linked immunosorbent assay (ELISA). Each lysate antigen was diluted (2000 ng of protein/mL) in a carbonate-bicarbonate coating buffer (pH 9.6) and then added to triplicate wells (100 uL) of a NUNC 96-well microplate (Fisher-Thermo). The plates were then incubated overnight at 4°C in a humid chamber followed by washing three times with phosphate buffered saline containing 0.15% Tween 20 (PBS-T). The serum specimens (1: 2500 dilution; 100 uL) were added to the microplate wells and incubated for 30 min at 37°C in a humid chamber. Following this incubation the wells were washed as above and 100 uL of goat anti-dog IgG (H & L) peroxidase conjugate (Kirkegaard and Perry, Gaithersburg, MD, USA) was added to each well and incubated for 30 min at 37°C. The plates were again washed as above and 100 uL of each of the 3 TMB peroxidase substrates (1-step Ultra TMB-ELISA, Thermo Scientific (Rockford, IL, USA), and SureBlue TMB Microwell Peroxidase (1-component); SureBlue Reserve TMB Microwell Peroxidase (1-component), KPL (Gaithersburg, MD, USA)) was added to the wells and incubated for approximately 3 min at room temperature. The first reaction was stopped by the addition of sulfuric acid and the other two reactions were stopped by TMB Stop Solution (KPL). The absorbance was then read at 450 nm using a BIO-RAD 2550 EIA reader.

### 2.4. Critical Absorbance Value

In order to determine the detection limit of the assay, we determined the critical absorbance value (which designates positive versus negative results) which was calculated by adding three times the standard deviation (SD) of the normal serum specimens to the mean absorbance value of the normal specimens. Three times the SD is approximately equal to the 99% confidence level.

## 3. Results and Discussion

The 4 lysate antigens were compared for antibody detection in the 10 serum specimens from the normal, noninfected dogs. Absorbance values ranged from 0.242 to 0.369 (B5896; mean absorbance value: 0.284; critical absorbance value: 0.353), from 0.221 to 0.356 (ERC-2; mean absorbance value: 0.280; critical absorbance value: 0.349), from 0.246 to 0.373 (B5931; mean absorbance value: 0.303; critical absorbance value: 0.372), and from 0.247 to 0.385 (T-58; mean absorbance value: 0.314; critical absorbance value: 0.383). Thus any absorbance value above the critical value is equal to antibody detection at the 99% confidence level.

**Figure 1:**
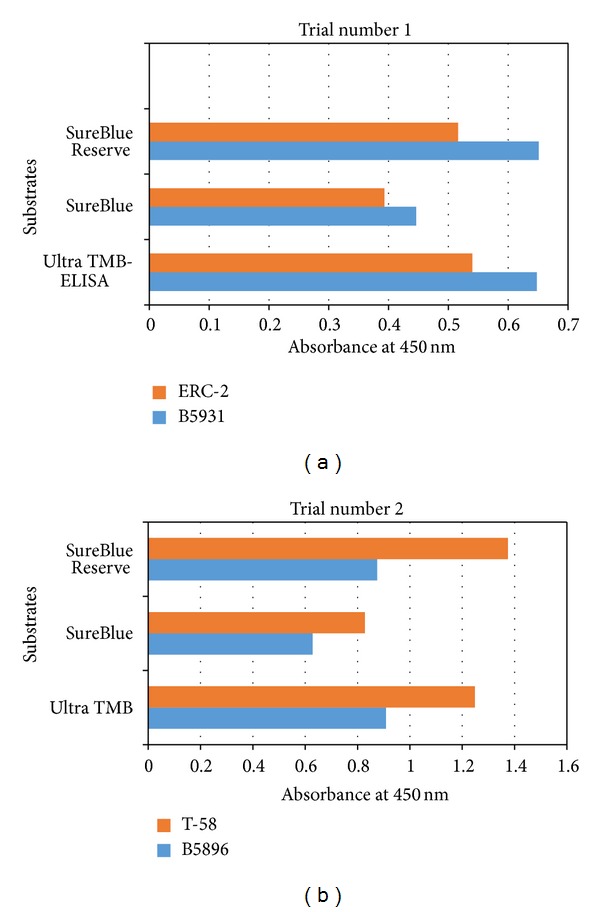
Comparison of the mean absorbance values of lysate antigens prepared from dog (ERC-2, T-58) and human (B5931, B5896) isolates using three different substrates (Ultra TMB, SureBlue, and SureBlue Reserve) for antibody detection in the 48 serum specimens from dogs with blastomycosis (24 for each trial: Trial 1 and Trial 2).

The 4 lysate antigens in combination with the 3 substrates all resulted in mean absorbance values (24 specimens; Trials 1 and 2) greater than the critical absorbance value (an indicator of positive versus negative detection) for each antigen as shown in Figure 1. In Trial 1, the B5896 lysate antigen (human isolate) proved to be more reactive than the lysate prepared from the dog isolate (ERC-2) with mean absorbance values ranging from 0.446 to 0.651. The Ultra TMB and the SureBlue Reserve substrates exhibited comparable reactivity in Trial 1.

In Trial 2, the T-58 lysate preparation from the dog isolate showed greater reactivity than the lysate prepared from the human isolate (B5896) with mean absorbance values ranging from 0.628 to 0.909. The greatest amount of reactivity was achieved with the T-58 antigen and the SureBlue Reserve substrate.

## 4. Conclusion

Our study, which compared 4 lysate antigens and 3 peroxidase substrates with regard to antibody detection in sera from dogs with blastomycosis, indicated that the optimal *B. dermatitidis* antigenic preparation was the lysate prepared from the dog isolate T-58 and used in combination with the KPL SureBlue Reserve substrate in the indirect ELISA. As KPL indicates in their advertisements, their top tier SureBlue Reserve is the substrate of choice when the highest sensitivity is required.
